# The Nutrition and Health in Southwest China (NHSC) study: design, implementation, and major findings

**DOI:** 10.1038/s41430-020-00703-6

**Published:** 2020-08-15

**Authors:** Xiao Zhang, Mengxue Chen, Ruonan Duan, Hongmei Xue, Jiao Luo, Xiaohua Lv, Hong Jia, Fang He, Lishi Zhang, Guo Cheng

**Affiliations:** 1 West China School of Public Health and West China Fourth Hospital, Sichuan University, Chengdu, PR China; 2 West China School of Public Health and Healthy Food Evaluation Research Center, Sichuan University, Chengdu, PR China; 3 College of Public Health, Hebei University, Baoding, PR China; 4 Department of Epidemiology and Biostatistics, School of Public Health, Southwest Medical University, Luzhou, PR China; 5 Laboratory of Molecular Translational Medicine, Center for Translational Medicine, Key Laboratory of Birth Defects and Related Diseases of Women and Children (Sichuan University), Ministry of Education, Department of Pediatrics, West China Second University Hospital, Sichuan University, Chengdu, Sichuan PR China; 6 West China School of Nursing, Sichuan University, Chengdu, Sichuan PR China

**Keywords:** Epidemiology, Nutrition

## Abstract

**Background:**

There are few studies of nutritional and genetic factors and their interactions on the risk of noncommunicable diseases (NCDs) among Chinese adults.

**Objective:**

Our aim for the Nutrition and Health in Southwest China (NHSC) study is to investigate the impact of diet, lifestyle, genetic background, and their interactions on NCDs among adults in Southwest China.

**Methods:**

The NSHC is a prospective cohort study initiated in winter 2013. The baseline data collection was completed in December 2018, and follow-ups are conducted every 2 years. Information on genomics, anthropometry, nutrition, eating behaviors, physical activity, depression and mental disorders, clinical and biochemical examinations, and lifestyles was collected.

**Results:**

7926 adults completed the baseline questionnaire. The average age of participants was 42.6 (9.8) years at study enrollment. More than half were female, 37.2% had achieved more than 12 years of education, and 49.3% of them came from family income >35,000 Yuan. Our analyses of the baseline data suggested that adults with greater dietary energy density appeared to have greater body mass index, fat mass index, fat-free mass index and percentage body fat, and that participants with a higher level of dietary glycemic index, glycemic load, or serum 25(OH)D had a less favorable glucose homeostasis. In addition, spending less time watching television and having a healthy eating pattern may play significant roles in later cellular aging.

**Conclusions:**

In conclusion, the NHSC cohort provides valuable data for investigations of the relevance of gene, nutrition, lifestyles, and their interactions on NCDs among southwestern Chinese adults.

## Introduction

As urbanization and westernization increase, China is facing great challenges from NCDs [1, 2]. A better knowledge of the contribution of nutrition, lifestyle, and genetic susceptibility to NCDs among Chinese is thus of public health relevance. It has been suggested that unhealthy diet and lifestyle play a significant role in the development of NCDs [3]. Compared to western populations, Asians appear to have a greater genetic predisposition to T2DM [4, 5]. These ethnic differences in the associations of risk factors with NCDs may result from differences in genetic susceptibility to NCDs or from interactions between diet, lifestyle, and genetic background. There are few epidemiological studies investigating environmental factors, genetic factors, and their interactions on the risk of NCDs in China. To fill this gap, the Nutrition and Health in Southwest China (NHSC) study was initiated, with the intention of investigating the impact of diet, lifestyle, genetic background, and their interactions on obesity, diabetes, hypertension, cardiovascular diseases, and other NCDs among Chinese adults.

## Design and methods

### Study design and overview

**Fig. 1:**
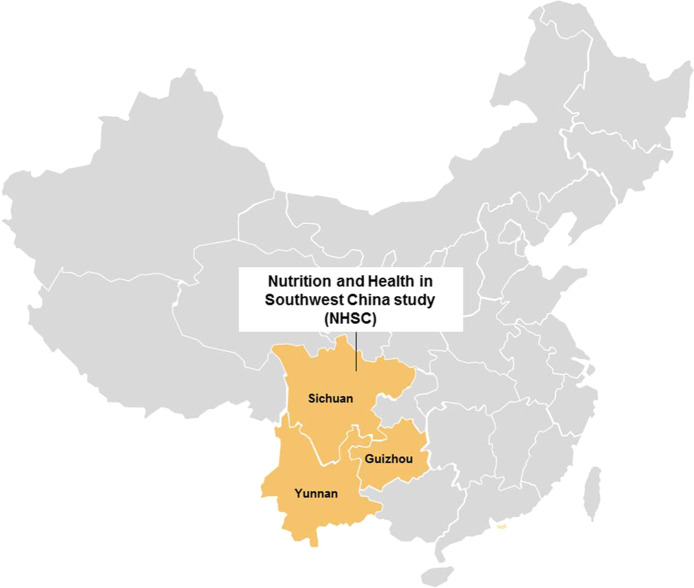
Location of the Nutrition and Health in Southwest China (NHSC) study. Including Sichuan, Guizhou and Yunnan provinces.

The NSHC is a prospective cohort study initiated in winter 2013. Using a sampling design stratified by urban and rural locations, a representative sample of Chinese adults was drawn from the general population in Southwest China (0.49 million sq.km, 144.1 million residents [6], Fig. 1). In total, 54 study sites (23 communities and 31 villages) were included as of December 2018. At each site, a two-stage (household-person) sampling method was used. Of the households listed within each site by the resident offices, 150 were randomly selected. Eligible participants were adults aged 18–70 years to facilitate follow-up, only persons who had lived in their current residence for at least 1 year were eligible to participate. When a member of the household refused participation or was unavailable, a replacement household was selected using a simple random sampling method from all households of similar composition in the same site, excluding the already selected households. The replacement households were recruited to ensure an adequate sample size within each selected site; they also helped to maximize the representativeness of the surveyed communities with regard to the prevalence of major chronic diseases as well as the distributions of age, gender, educational, and individual economic status. The study was approved by the Ethics Committee of Sichuan University. All participants provided written informed consent for the examinations.

**Fig. 2:**
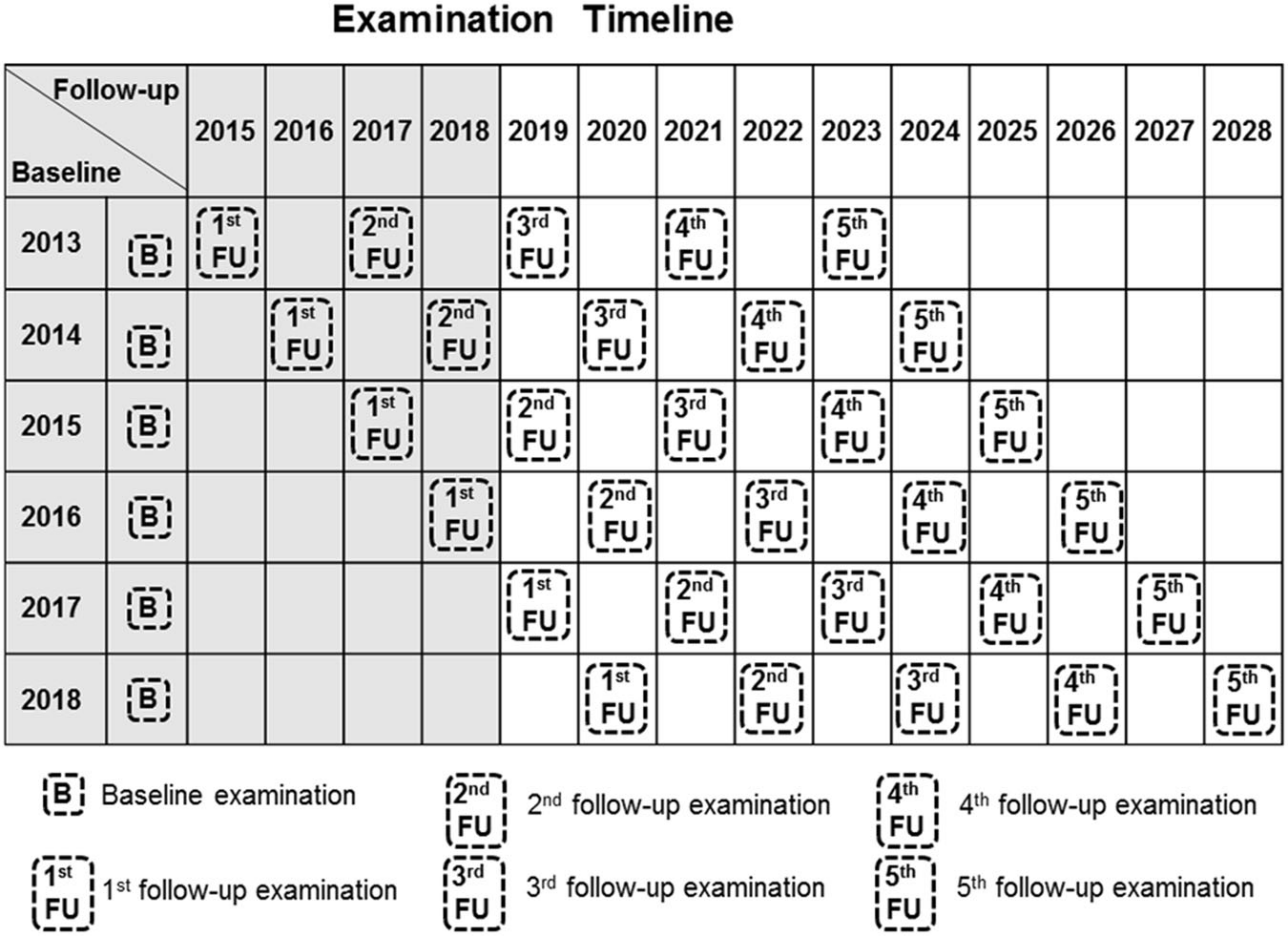
Examination timeline of the Nutrition and Health in Southwest China study. The baseline data collection was from 2013 to 2018 and follow-ups are conducted every 2 years.

The baseline data collection was completed in December 2018 and follow-ups are conducted every 2 years. Figure 2 displays an overview of the study protocol and examination timeline. Up to now, at least one follow-up assessment has been completed for participants recruited in 2013, 2014, 2015, and 2016, with two follow-ups completed for participants recruited in 2013 and 2014.

### Data assessment

All assessments of participants in the NHSC Study have included (and continue to include) questionnaires, anthropometric measurements, medical examinations and biochemical measurements. Table 1 shows a list of exposure and outcome variables available from the NHSC study instruments.

**Table 1:**
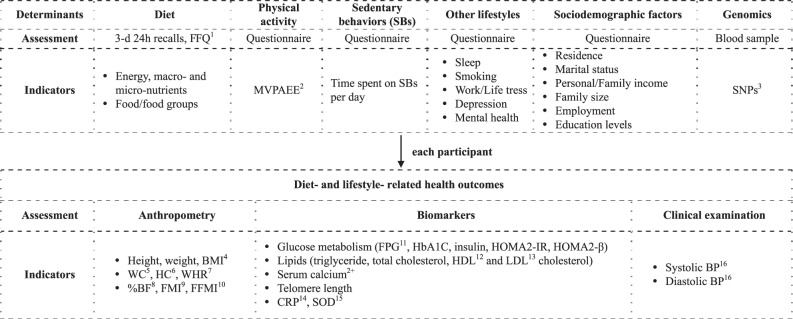
Design and major components of the Nutrition and Health in Southwest China study.

^a^FFQ, food frequency questionnaire.

^b^MVPA energy expenditure, energy expended on moderate-to-vigorous physical activities (MJ/day).

^c^SNPs, single nucleotide polymorphisms.

^d^BMI, body mass index.

^e^WC, waist circumference.

^f^HC, hip circumference.

^g^WHR, waist to hip ratio.

^h^%BF, percentage body fat.

^i^FMI, fat mass index.

^j^FFMI, fat-free mass index.

^k^FPG, fasting plasma glucose.

^l^HDL, high density lipoprotein.

^m^LDL, low density lipoprotein.

^n^CRP, C-reactive protein.

^o^SOD, superoxide dismutase.

^p^BP, blood pressure.

### Nutrition assessment

Dietary intake data are collected via three 24-h recalls and a food frequency questionnaire (FFQ). The 24-h recalls were obtained by trained investigators in face-to-face interviews first on the day of registration and subsequently on two other days (selected by the participants) within a 10-day period from the registration day. For 24-h recalls, details on recipes as well as the types and brands of all food items reported were obtained. Dietary intake data from the 24-h recalls were converted into nutrient intake data using the continuously updated in-house nutrient database, which was based on the China Food Composition [7].

Participants’ consumption of foods and food groups over the last year was collected via a 66-item interviewer-administered FFQ [8]. The frequency values of FFQ range from never consumed to consumed more than five times per day using the standard serving size for food groups. Nutrient intakes were then calculated by multiplying the frequency of consumption of each food or beverage by the nutrient content of the portion and summing the intake of each nutrient for all items.

### Eating behaviors

Eating behaviors relevant to diet quality of the participants were obtained by questionnaire. Participants were interviewed about the number of times per week they ate breakfast, time and place they ate breakfast, and foods commonly eaten for breakfast. Similar details (time, location, commonly eaten foods) were obtained for lunch and dinner. In addition, participants were interviewed concerning the frequency of having dinner with family, the frequency of snacking and food type of snacks, and the frequency of eating outside the home. Moreover, the local diet specialties were considered.

### Physical activity

Level of physical activity was assessed by a physical activity questionnaire with 38 items validated for Chinese adults [9]. Participants were asked the usual type, frequency and duration of activities over the past 12 months. Participants are also asked “What kind of transportation do you usually use when you go to work?”. The frequency and duration of sedentary behaviors were also obtained, including watching television, using computers, using smart phone, reading, and playing cards or mahjong.

### Lifestyle-related information

Participants were asked to report their smoking habits. For the ex-smokers and current smokers, number of cigarettes smoked per day and duration of cigarette smoking in years were also collected.

The sleep duration and sleep quality of the participants were assessed using the Pittsburgh sleep quality index scale, in which 19 individual items generate 7 component scores [10]. In addition, the frequency and duration of napping were obtained.

The assessments of work and life stress were based on the participants’ subjective rating of work and life stress. Participants are asked “how often do you feel stressed at work/in daily life?” and the level of stress they felt. The response options for the participant’s level of stress included not at all stressful, not very stressful, a bit stressful, quite a bit stressful, and extremely stressful.

### Depression and mental disorders

Since 2015, the depression status of the participants has been assessed by trained investigators, using the 17-item Hamilton Rating Scale for Depression (HDRS-17) [11]. The Chinese version of HDRS-17 has been shown to have acceptable validity and reliability [12]. In addition, information on mental health status has been collected since 2017, using the Mini-Mental State Examination [13].

### Anthropometric measurements

All anthropometric measurements were performed by trained investigators with the participants dressed lightly and barefoot. Height and weight were measured to the nearest 0.1 cm and 0.1 kg, respectively, using an Ultrasonic Weight and Height Instrument (DHM-30, Dingheng Ltd, Zhengzhou, China). Waist circumference was measured at a point midway between the lowest rib margin and the iliac crest in a horizontal plane using a nonelastic tape. Hip circumference was measured to the nearest 0.1 cm at the maximal gluteal protrusion. Skinfold thicknesses were measured on the right side of the body to the nearest 0.1 mm using a Holtain caliper (Holtain Ltd., Crosswell, United Kingdom). All anthropometric measurements were performed twice for each participant.

### Blood pressure

After a 5–10 min rest in a quiet environment, blood pressure was measured twice using a mercury sphygmomanometer on the right upper arm of participants. Systolic and diastolic blood pressures were recorded for each participant.

### Biochemical measurements

Blood samples were drawn after an overnight fast of at least 10 h for all participants at every visit. Venous blood specimens were collected using vacuum blood collection tubes containing anticoagulant sodium fluoride or ethylenediaminetetraacetic acid. Blood samples were centrifuged and stored at 4 °C for subsequent analysis. The biochemical assessments are shown in Table 1.

### Genomics

Genomic DNA was extracted from peripheral blood lymphocytes using standard methods. The stock samples were split into five tubes and stored at different locations at −80 °C. DNA samples were genotyped using the Infinium II technology from Illumina (Human HAP300 panel).

### Additional information

The sociodemographic characteristics were obtained via a questionnaire: including age, place of residence, marital status, family size, personal income per month, family income per year, years of education, and employment status. Family history of chronic diseases as well as the timing of menarche and menopause was also collected.

### Quality assurance procedures

Prior to study implementation, a 3-day training was conducted at the study offices of each site. An operation manual was developed detailing all standardized procedures. To improve the accuracy of the estimated portion sizes during dietary assessment, standard serving bowls, plates and glasses, and a photo book containing photos of snacks and beverages were displayed to the participants. Finally, to encourage participants to complete the examination, each of them was given the results of their clinical examination at no charge within a week after the visit.

## Results

### Participants’ characteristics

In total, as of 2018, 8612 adults were invited to participate in the NHSC study. Of these, 686 declined due to long duration or no interest (overall response rate 92%); 7926 adults completed the baseline questionnaire. Baseline characteristics for the entire cohort are shown in Table 2.

**Table 2:** Baseline characteristics^a^ of 7926 adults aged 18–70 years in the Nutrition and Health in Southwest China study.

Characteristics	Value
Age, years	42.6 (9.8)
Female, %	53.9
Urban residence, %	31.2
>12 years of education^b^, %	2948 (37.2)
Family income >35,000 Yuan^c^, %	3908 (49.3)
Nutritional data	
Energy intake, kcal/d	1997 (525)
Protein, % of energy	16.6 (4.3)
Fat, % of energy	30.6 (5.9)
Carbohydrate, % of energy	52.8 (12.7)
Lifestyle data	
MVPA energy expenditure^d^ (MJ/day)	2.7 (1.9)
Sleep duration ≥7 h/d and ≤9 h/d^e^, %	4978 (62.8)
Current smoker, %	1712 (21.6)
Anthropometry	
Body mass index, kg/m^2^	23.3 (2.7)
Overweight, %	3305 (41.7)
Waist circumstance (cm)	85.7 (9.6)
Percentage body fat, %	35.1 (31.2, 42.6)
Biochemical measurements	
Fasting plasma glucose, mmol/L	5.3 (4.6, 5.7)
Glycated hemoglobin, %	5.5 (5.1, 5.8)
Insulin, μIU/mL	6.2 (4.9, 9.8)
HOMA2-IR^f^	0.9 (0.6, 1.5)
Prediabetes^g^, %	3138 (39.6)
Systolic blood pressure, mmHg	125.2 (15.6)
Diastolic blood pressure, mmHg	75.3 (17.1)
Total cholesterol, mg/dL	4.5 (4.1,5.6)
High-density lipoprotein cholesterol, mg/dL	1.2 (1.1,1.5)
Low-density lipoprotein cholesterol, mg/dL	3.2 (2.8,3.8)
Triglyceride, mg/dL	1.2 (0.8,1.8)
C-reactive protein, mg/L	3.0 (1.3, 5.2)
Superoxide dismutase, U/mL	146.1 (19.2)
Serum calcium^2+^, mmol/L	2.4 (2.2, 2.5)
Serum 25(OH)D, ng/mL	20.6 (15.0, 27.8)

^a^Values are means (SD), medians (Q1, Q3) or frequencies.

^b^At least 12 years of school education.

^c^Average family income more than 35,000 Yuan every year.

^d^MVPA energy expenditure, energy expended on moderate-to-vigorous physical activities (MJ/day).

^e^Recommended sleep duration for adults according to the National Sleep Foundation [14].

^f^HOMA2-IR, Homeostasis model assessment 2: insulin resistance, calculated by Wallace Formula [22].

^g^Pre-diabetes was defined using the updated classification and diagnosis of diabetes of American Diabetes Association (ADA) [3].

### Findings to date

Up to now, six articles reporting on the NHSC baseline data have been published [14–19]. Different exposures have been investigated, and body composition and glucose homeostasis have been examined; the key findings are summarized in Table 3.

**Table 3:** Major findings from the Nutrition and Health in Southwest China study.

Publication	Major concern	Main findings
Yin et al. [14]	Dietary energy density and body composition	Higher dietary energy density (ED) appeared to have higher body mass index, fat mass index, fat-free mass index and percentage body fat. Furthermore, ED contributed to higher increases of body composition in women than in men.
Chen et al. [15]	Nocturnal sleep duration, midday nap duration and body composition	Nocturnal sleep duration was inversely associated with body mass index, waist circumference, percentage body fat and fat mass index in men, but not in women; additionally, midday nap <1 h was significantly related with the lower prevalence of overweight in both men and women.
Cheng et al. [16]	Dietary carbohydrate quality and glucose homeostasis	Higher dietary glycemic index or glycemic load was related to a less favorable glucose homeostasis. Furthermore, these adverse associations were particularly pronounced among those with an increased genetic predisposition towards T2DM or those with a low cereal fiber intake.
Li et al. [17]	Serum 25(OH)D level and risk of impairment of glucose homeostasis	A higher serum 25(OH)D level is associated with decreased risk of impairment of glucose homeostasis among adults without diabetes in Southwest China.
Xue et al. [18]	Television watching and telomere length	Television watching is inversely related to telomere length among Chinese adults.
Gong et al. [19]	Dietary pattern and telomere length	Higher adherence to the ‘vegetable-rich’ dietary pattern is related to longer telomere length in Chinese women. Furthermore, this relationship was partially explained by C-reactive protein but not by body fat.

### Diet, lifestyle, and body composition

Data from 1933 adults indicated that a greater dietary energy density was associated with greater body mass index (BMI), fat mass index (FMI), fat-free mass index and percentage body fat (PBF) [14]. These associations were more relevant in women than in men.

Using data from 3145 adults, nocturnal sleep duration was found to be inversely associated with BMI, waist circumference, PBF, and FMI in men, but not in women; a midday nap of <1 h duration was significantly associated with a lower prevalence of overweight in both men and women [15].

### Diet, serum 25(OH)D, and glucose homeostasis

A greater dietary glycemic index value or glycemic load was found to be related to a less favorable glucose homeostasis in 1514 Chinese adults [16], which were particularly pronounced among those with an increased genetic predisposition towards T2DM or those with a low cereal fiber intake. Moreover, a higher serum 25(OH)D level was associated with a lower risk of impairment of glucose homeostasis among adults without diabetes [17].

### Diet, lifestyle, and cellular aging

Telomere length shortening is considered a potential indicator of cellular aging. Television watching was shown to be inversely associated with telomere length [18]; greater adherence to a “vegetable-rich” dietary pattern was related to longer telomere length in women [19].

## Discussion

The NHSC is a population-based cohort study in China, which prospectively addresses research questions concerning the impact of interactions between diet, lifestyles and genetic susceptibility on health outcomes of Chinese adults. As it is a population-based cohort study, the NHSC enables us to further understand the determinants of NCDs.

The NHSC is a longitudinal open cohort study with broad research aims. The major advantages include its random sampling with a relatively large sample size, long duration of follow-up, detailed and repeated assessments of sociodemographical characteristics, dietary intake, and lifestyle practices as well as the use of comprehensive biochemical markers to characterize health outcomes. A further advantage lies in the inclusion of genetic data, as objective indicators can be used to characterize the genetic background of the participants.

Several limitations should be mentioned. First, attrition is a concern. Although we have made efforts to retain cohort participants, the response rates have decreased over time in common with most cohort studies. Second, as people who are concerned with their health are more likely to participate in the study, we could not rule out the possibility of healthy volunteer bias.

In summary, the NHSC study has provided valuable data not only with regard to indicators of health outcomes but also on diet, lifestyle and genetic factors. The NHSC study is expected to determine the interactions of these factors in the development of NCDs and to provide further insights into the underlying mechanisms.

## Disclaimer

The interpretation and reporting of these data are the sole responsibility of the authors.
